# AI-driven parametrization of Michaelis–Menten maximal velocity: Advancing in silico new approach methodologies (NAMs)

**DOI:** 10.1016/j.namjnl.2025.100012

**Published:** 2025-02-14

**Authors:** Achilleas Karakoltzidis, Spyros P. Karakitsios, Dimosthenis Α. Sarigiannis

**Affiliations:** a Aristotle University of Thessaloniki, Department of Chemical Engineering, Environmental Engineering Laboratory, University Campus, Thessaloniki 54124, Greece; b HERACLES Research Center on the Exposome and Health, Center for Interdisciplinary Research and Innovation, Balkan Center, Bldg. B, 10th km Thessaloniki – Thermi Road, 57001, Greece; c EnvE.X, K. Palama 11, Thessaloniki, Greece; d School for Advanced Study (IUSS), Science, Technology and Society Department, Environmental Health Engineering, Piazza della Vittoria 15, Pavia 27100, Italy; e National Hellenic Research Foundation, Athens, Greece

**Keywords:** Deep learning, *V*_max_, Maximal velocity, Enzyme structure, QSPR, NAMs

## Abstract

The development of mechanistic systems biology models necessitates the utilization of numerous kinetic parameters once the enzymatic mode of action has been identified. Simultaneously, wet lab experimentation is associated with particularly high costs, does not adhere to principles of reducing the number of animal tests, and is a time-consuming procedure. Alternatively, an artificial intelligence-based method is proposed that utilizes enzyme amino acid structures as input data. This method combines NLP techniques with molecular fingerprints of the catalysed reaction to determine Michaelis–Menten maximal velocities (*V*_max_). The molecular fingerprints employed include RCDK standard fingerprints (1024 bits), MACCS keys (166 bits), PubChem fingerprints (881 bits), and E-States fingerprints (79 bits). These were integrated to produce reaction fingerprints. The data entries were sourced from SABIO RK, providing a concrete framework to support training procedures. After the data preprocessing stage, the dataset was randomly split into the training set (70 %), validation set (10 %), and test set (20 %) ensuring unique amino acid sequences for each subset. The data points with structures similar to the ones used to train the model as well as uncommon reactions were employed to further test the model. The developed models were optimized during the training procedure to predict *V*_max_ values efficiently and reliably. Utilizing a fully connected neural network, these models can be applied to all organisms. Amino acid proportions of enzymes were also tested resulting in an unreliable predictor for the *V*_max_ value. During testing, the model demonstrated better performance on known structures compared to unseen data. In the given use case, the model trained solely on enzyme representations achieved an *R*-squared of 0.45 on unseen data and 0.70 on known structures. When enzyme representations were integrated with RCDK fingerprints, the model achieved an *R-squared* of 0.46 on unseen data and 0.62 on known structures.

## 1 Introduction

The Michaelis and Menten (1913) approach is widely used for the determination of enzyme kinetics. Such a system is simply described by the chemical (1) and mathematical (2) equations below:

(1) E+S⇌ES→E+P

(2) $$v=\;\frac{V_{\text{max}}\left[S\right]}{K_{m}+\left[S\right]}$$

*V*_max_ (maximal velocity) represents the maximum rate or velocity of an enzyme-catalysed reaction when the enzyme is completely saturated with its substrate. It serves as a fundamental characteristic of a specific enzyme under defined conditions of enzyme concentration, temperature, and pH (Ahenkorah et al., 2021) and it is influenced by both the enzymatic concentration and structure. A quantitative understanding of interactions between enzymes and metabolites relies on the determination of *V*_max_ values. Given that, the metabolome and systems biology philosophy are bridged with cellular biology by linking the intracellular concentration of a metabolite to the rate of its consumption.

However, the experimental estimation of turnover numbers and Michaelis–Menten constants presents numerous challenges and complexities. Wet lab experiments often necessitate specialized infrastructure, skilled personnel, and expensive reagents, which may pose barriers for most of the researchers especially when resources are limited. Furthermore, the maintenance and calibration of equipment contribute to the overall costs of conducting such experiments. In addition, many wet lab procedures are also time-consuming, involving multiple steps and lengthy incubation periods. It is often invested significant time and effort into optimizing protocols and executing experiments, which potentially impede research progress. Additionally, working with live organisms or human samples in wet lab settings raises ethical concerns, particularly concerning animal welfare and the use of genetically modified organisms. Ethical approval procedures are also time consuming depending on the bureaucracy that each organization committee has. The stringent regulations and guidelines must be adhered to ensure the ethical treatment of experiments. Last but not least, reproducing wet lab experiments can also be challenging due to factors such as variability in biological samples, environmental conditions, equipment performance, or experimental techniques. Indeed, given the challenges associated with wet lab experiments, the development of alternative methodologies is imperative to improve research efficiency and gradually replace traditional experimental procedures in the biomedical research and beyond.

In contrast, Artificial Intelligence (AI) methodologies enable the rapid prediction of key metabolic parameters, such as *V*_max_, directly from structure-based information, including the enzyme structure and interacting metabolites. This approach not only eliminates the need for unnecessary experimental procedures but also captures the complex, non-linear relationships inherent in cellular metabolism. Simultaneously, AI methodologies are gaining increasing popularity across numerous scientific disciplines due to their multiple applications and ability to produce reliable results. One established approach of applying AI concerns the parameterization of kinetic models in exposure biology through the utilization of QSARs (Quantitative Structure Activity Relationships). The work of Papadaki et al. (2017) and Sarigiannis et al. (2017) are notable examples in this area. They have developed Artificial Neural Network (ANN) models designed to predict the partitioning of substances into human organs such as kidneys, heart, liver, brain, lungs etc. as well as models that are focused on estimating two key metabolic parameters: the bodyweight-normalized maximal velocity and the Michaelis–Menten constant (*K*_m_) for a plethora of environmental chemicals. A detailed workflow of how advanced AI models can support risk assessment is presented in the work of Sarigiannis et al. (2020).

Further examples of AI applications include the estimation of protein binding (Alipanahi et al., 2015; Ballester and Mitchell, 2010; Evteev et al., 2023; Sehnem Heck et al., 2017), RNA binding estimates (Yazdani et al., 2023), detection of primary regulatory genes, and genes controlling transcriptional processes associated with cancer (Califano and Alvarez, 2017; Carro et al., 2010; Krempel et al., 2018). Additionally, AI has been used to describe the structure and dynamics of networks controlling gene expression at the transcriptional level (Chauhan et al., 2021; Djebali et al., 2012; Marbach et al., 2012) as well as overall gene expression (Zrimec et al., 2020; Zrimec et al., 2019), genome annotation (Leung et al., 2015; Yip et al., 2013), identification of plant-pathogen interactions (Mishra et al., 2019), and forecasting the metabolic roles and activities within intricate communities of microorganisms (Colarusso et al., 2021; Dutta et al., 2022; Kang et al., 2022; Langille et al., 2013). It is also noteworthy to mention approaches where AI models are trained on in silico data producing trustworthy results, with numerous instances documented in the literature (Chou and Lin, 2023; Khan et al., 2023; Sulaiman et al., 2023a; Sulaiman et al., 2023b; Sulaiman and Khan, 2023; Sulaiman et al., 2023c). It is obvious that any task described by a reliable set of data provides patterns which can be learned and then applied to unseen datasets. This significant benefit lies in the capability of machine learning (ML) techniques to sort through extensive datasets to uncover patterns that might otherwise go unnoticed (Camacho et al., 2018).

Considering the widespread acceptance of AI methodologies in the field of computational biology, the determination of enzyme kinetics emerges as one of the most prominent applications. From this perspective multiple AI models were identified for the estimation of Michaelis–Menten *K*_m_ (Borger et al., 2006; Kroll et al., 2021; Yan et al., 2012) as well as AI models for turnover (*k*_cat_) numbers (Heckmann et al., 2018; Kroll et al., 2022a; Li et al., 2022) but no models for the determination of *V*_max_ has been reported so far. One potential reason for this variability is the non-stationary nature of the anticipated value for *V*_max_ in a cellular system, as well as its strong dependence on the bioavailable concentration. Consequently, in vitro estimation of *V*_max_ is straightforward, as AI models are available to approximate *k*_cat_ values, and the determination of an enzyme concentration is relatively uncomplicated. However, the estimation of *V*_max_ cannot solely rely on in vitro systems, especially within the regulatory arena of risk assessment. Considering the 3Rs (Replacement, Reduction, and Refinement) principles, along with the increasing adoption of computational approaches as alternatives to experimentation, the introduction of computational New Approach Methodologies (NAMs) is imperative. NAMs have recently emerged in response to the green transition in the EU and the targets set by the European Commission's chemicals strategy for sustainability until 2050 (Apel et al., 2023; Doak et al., 2022; Karakoltzidis et al., 2024).

In the present study, a deep learning model is introduced with the aim of precisely estimating the Michaelis–Menten maximal velocity. This novel tool complements computationally evolving mechanistic models and NAMs by not only estimating *V*_max_ but also promoting the adoption of a holistic in silico approach for developing deterministic systems biology models. Combining this model with existing literature models (e.g., *k*_cat_ models) enables the determination and initialization of all kinetic parameters for complex biology-based deterministic models. As a result of that, it is now possible to exclusively determine the kinetics of complex mathematical models by employing only AI methodologies (*k*_cat_, *K*_m_, *V*_max_). As already mentioned, to the best of our knowledge, this is the first model to approximate Michaelis–Menten maximal velocity as well as the first application of TensorFlow and Keras architectures for the derivation of enzyme kinetics.

Moreover, the application of the current model enables the estimation of cellular enzyme concentrations in in-vivo applications and can significantly enhance the complexity and understanding of interrelationships in biological systems, all without the need for experimental procedures. Furthermore, such methodologies allow the potential link between transcriptional processes with metabolic ones. The deep learning model developed can serve as a key component in a computational methodology for developing robust mechanistic systems biology models, without relying on kinetic data from laboratory experiments, but instead utilizing information from existing literature. Moreover, appropriate, and reliable In Vivo In Vitro Extrapolation (IVIVE) models can be employed to transform the predicted values from the model into human-scale or other in vivo model mappings. As a result of that, these values have broad confidence intervals and exhibit limited correlation with experimentally observed values (Khodayari and Maranas, 2016). Hence, the ability of artificial intelligence to forecast these parameters, within a wide range and with a high degree of uncertainty, would signify a meaningful advancement toward creating more precise and robust representations of cellular metabolism. This, in turn, contributes to the development of in silico NAMs for risk assessment.

## 2 Methodological framework and data pre—processing

### 2.1 Software

Code was implemented in R 4.2.2 (R Developement Core Team, 2009) and Python 3.9.0 (Sanner, 1999) in a Windows 11 desktop PC with the GPU NVIDIA 2070 Ti. The Integrated Development Environments (IDE) that were used were R Studio 2023.03.0386 and PyCharm 2021.2.2 (Professional Edition). R and Python are widely used as statistical computing languages for their robust data analysis capabilities and AI applications. Artificial Neural Networks (ANN) were implemented using the deep learning libraries TensorFlow and Keras (Pang et al., 2020) in the R environment. TensorFlow, developed by Google, is a leading open-source deep learning library that provides a flexible and scalable platform for building complex neural networks of multiple architectures. Keras, built on top of TensorFlow, offers a high-level API, making it user-friendly and efficient for constructing deep learning models. Enzyme representations were generated using the Python programming environment.

The graphs have been introduced using ggplot2 (Wickham, 2011) in the R computing environment (R Developement Core Team, 2009). Statistical analysis of predictions with the experimentally measured values has been conducted in the R computing environment as well. Equations have been integrated into the graphs using the ggpmisc library (Aphalo, 2016).

### 2.2 Data collection and data cleaning

The construction of a concrete artificial intelligence model depends on the data availability and the data quality. Given the high uncertainty on the determination of *V*_max_ values we chose to use only data originated from Sabio RK (Wittig et al., 2012) and more specifically only information concerning wild enzymes. Data from SABIO RK was manually collected due to the unavailability of a related library providing an interface to an API (Application Programming Interface) in any programming language. This manual collection process yielded 1795 unique Excel files, each containing information for only one wild-type enzyme. To our knowledge, we downloaded all the available information that existed there. The raw excel files are also provided in the GitHub page.

All Excel files contained the same number of columns, simplifying integration into a common table. In order to acquire some computational time, parallel computing methodologies (Weston and Calaway, 2015) were also employed by incorporating the R-library doParallel to load and combine the raw excel files from SABIO RK. This consolidation resulted in a table with 1,236,908 rows. We then filtered the table to include only rows containing *V*_max_ values and removed any rows with missing values (NA), resulting in 858,351 rows. Additionally, we ensured that each row corresponded to only one enzyme by splitting rows with multiple enzymes and UniProt IDs. Rows where KEGG reaction ID or UniProt ID were not provided were removed. Duplicate data was eliminated as well. After completing the data cleaning stage of the SABIO RK dataset, the resulting table contained 43,633 unique rows.

To access reaction data from the KEGG database, it was necessary to organize all the data locally and make a single request for information, rather than continuous requests. This approach ensured faster execution of the algorithm since it eliminated the need to make repeated calls to the KEGG APIs, which could potentially introduce time constraints during the testing phase of the methodology. Specifically, all metabolic conversions (reactions), and endogenous metabolites were organized into two nested lists. Retrieving this information was achieved through communication with the KEGG APIs using the KEGGREST (Tenenbaum et al., 2019) and KEGGgraph (Zhang and Wiemann, 2009; Zhang et al., 2015a) libraries. All libraries mentioned in this step were employed within the R computing environment and are part of the Bioconductor software (Gentleman et al., 2004). The resulting datasets from KEGG database are also available on the GitHub page.

### 2.3 Introduction of amino acid sequences

The introduction of enzyme structural information relies on the UniProt ID provided in the information from SABIO. These IDs were used to retrieve amino acid (AA) sequences from the UniProt database (UniProt Consortium, 2019) which serves as a hub for numerous protein-related information, including structure. To retrieve this data, the R-library UniprotR (Soudy et al., 2020) provided by Bioconductor (Gentleman et al., 2004) was employed.

### 2.4 Deal with multiple *V*_max_ values for a unique structure and reaction

Since multiple *V*_max_ values may correspond to one enzyme reaction and structure, series lacking a KEGG reaction ID were removed, as previously mentioned. This step facilitated the conversion of substrate and product names to KEGG IDs, enabling the identification of additional duplicate references. Consequently, a dataset consisting of 4215 unique entries was obtained.

The present dataset was segmented into chunks based on the amino acid sequence, the respective organism, as well as the *V*_max_ unit. This segmentation process yielded a list of 1472 data frames. It is important to note that some data frames contained more than one row. Therefore, considering the potential presence of identical structures with similar *V*_max_ values in the test set, which could significantly impact the performance of the model, it was decided that in cases where there was more than one record in a data frame, the maximum and minimum values for *V*_max_ from all rows would be retained. Using these maximum and minimum values, 10,000 values were generated randomly, representing a uniform distribution. Using these maximum and minimum values, 10,000 values were randomly generated to represent a uniform distribution. For each distribution, the geometric mean was calculated, which was then utilized as the *V*_max_ value for the corresponding amino acid sequence. The same approach was applied in instances where only one row was provided, with maximum and minimum values for a *V*_max_ occurrence. However, this methodology was not applied in cases where there was a unique *V*_max_ value for each sequence and organism. As a result, the resulting table contains 1472 unique entries with unique sequences as well. Last but not least, since concentration levels were not provided from SABIO RK for all the enzymes considered thus far, it has been decided not to incorporate this characteristic of the enzymes in this analysis. Consequently, the model presented in the next sections will serve as a reference point for the estimation of enzyme concentrations. Additionally, if concentration was employed as a predictor, this model could not be used for such approximations.

To conclude, duplicated structures with reactions that are not included in the training set were organized in a dataset to further test the developing models on known structures. This dataset contained 47 rows.

### 2.5 Definition of molecular fingerprints and reaction fingerprints

Each substrate should have a .mol file that describes its structure. The integration of .sdf files in the methodology allows for the generation of chemical descriptors and fingerprints which can be easily incorporated in the artificial intelligence algorithms representing structures. Molecular fingerprints definition demands the use of 2D .mol files which have been collected manually from the KEGG database and CheBI database. For the conversion algorithm, the RCDK package was employed, which is a widely accepted R cheminformatics library (Guha and Cherto, 2017) that provides a plethora of fingerprint options as well as an advanced framework for chemicals analysis. If an .sdf file was not available from both databases, the corresponding was removed.

We chose four different molecular fingerprints to represent the molecular structures of the substrates employed in the approach presented here. The main selection criteria were based on two key factors: (a) the number of bits used to encode each fingerprint, which determines the level of structural information captured, and (b) the practical applicability of the fingerprints in cheminformatics, particularly their established use in molecular modelling and reaction studies.

As a result of that, each substrate is represented with the standard 1024-bit fingerprint provided by RCDK and has been used in multiple studies (Choi et al., 2020; Willighagen et al., 2017), the MACCS key an 166-bit fingerprint (Durant et al., 2002) which is one of the most commonly used fingerprint types, the PubChem, an 881-bit fingerprint (Kim et al., 2019) which has been applied in several studies (Bean et al., 2017; Bender et al., 2007; Dey et al., 2018; Dimitri and Lió, 2017; Jamal et al., 2017; Liu et al., 2012; Mizutani et al., 2012; Poleksic and Xie, 2018; Wang et al., 2019; Yamanishi et al., 2012; Zhang et al., 2015b; Zhou et al., 2015), and the E-state a 79-bit fingerprint introduced by Hall et al. (1991) that has been employed in numerous studies as well (Barigye et al., 2013; Elton et al., 2018; Floris et al., 2014).

**Fig. 1:**
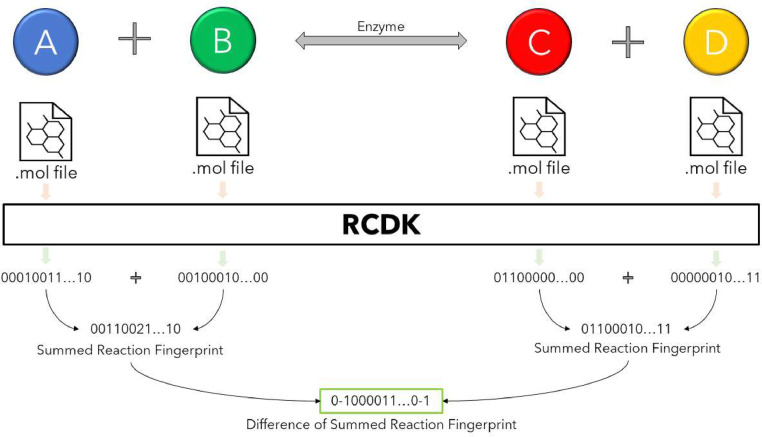
Introduction procedure of reaction fingerprints.

This diverse selection of molecular fingerprints enables a comprehensive analysis of substrate structures by balancing different levels of complexity and structural representation (e.g., number of bits). The resulting reaction fingerprint estimation relies on the work of Schneider et al. (2015) and the following Eq. (3) assuming that in this case agents are considered insignificant (4). The computational process to introduce a reaction fingerprint is described in detail in Fig. 1. A similar approach to concatenate individual molecular fingerprints and introduce reaction fingerprints has also been proposed by Kroll et al. (2023) and has been used for the other purposes such as clustering of chemical reactions (Probst et al., 2022). Finally, one of the key objectives of this study is to assess how the number of bits in a reaction fingerprint influences the amount of information captured about catalysed metabolic transformations within an artificial neural network framework. By comparing different fingerprint types, we aim to determine the significant level of structural encoding needed for accurate reaction modelling.

(3) $$reactionFP=\;w_{nonAgent}\left(\sum_{products}^{i=1}productFP_{i}-\;\sum_{reactants}^{i=1}reactantFP_{i}\right)+w_{agent}\sum_{agents}^{i=1}agentFP_{i}$$

(4) $$w_{nonAgent}=1,\;w_{agent}=0,\;\sum_{agents}^{i=1}agentFP_{i}=0$$

### 2.6 Introduction of ESM-1b representations

The methodology employed in this study to introduce ESM-1b vectors followed the approach proposed by Rives et al. (2021) and was subsequently adapted into a custom Python script to conform with our methodological framework. The original models and scripts from Rives et al. (2021) are available on GitHub. It is also important to note that the model utilizes a Transformers-based (Lin et al., 2022) architecture. Introducing protein language models that operate within an evolutionary context is a fundamental step towards advancing predictive and generative artificial intelligence methodologies for biological research. To achieve this, Rives et al. (2021) utilized unsupervised learning techniques to train a large language model (LLM). Their training set comprised a dataset containing 86 billion amino acids (AA) sourced from 250 million protein sequences, encompassing a diverse range of evolutionary contexts. This is the UniRef50 dataset and is available by the work of Suzek et al. (2015). The resulting model effectively captures significant biological insights within its representations, derived exclusively from sequence data. These representations encompass organizational structures that include information on the biochemical properties of amino acids, as well as protein homologies. Moreover, such a model encodes details about secondary and tertiary protein structures, which can be discerned through linear projections. More information about that can be found in the work of Rives et al. (2021).

The AA sequences retrieved from UniProt database (UniProt Consortium, 2019), as previously discussed in detail, were organized in a .fasta file format (Lipman and Pearson, 1985) by using the seqinR library (Charif et al., 2023) in the R computing environment. These sequences were then processed by the pretrained ESM-1b model to generate numerical protein representations. The resulting arrays, with specific dimensions of (1280, 1, 1), will be referred to as ESM-1b representations from now on.

The adopted methodology relies on the NLP (Natural Language Processing) modelling framework. The advantage of using such a tool regards the transition of all the words in a sentence being converted to numerical vectors that encode significant information about the content and the position of a word. The application of these models to amino acid (AA) sequences which contain the biochemical information for proteins, replaces the word-entity in normal NLP paradigms and takes advantage of it to create unique numerical representations based on the enzyme structure (Kroll et al., 2022a). As the number of entries in the training datasets increases the capabilities of such models learning representations is significantly improved (Baevski et al., 2019; Radford et al., 2019; Rives et al., 2021). Deciphering the information embedded within protein sequence variation has long been a challenge in computational biology. The approach applied not only by Rives et al. (2021) but also by Alley et al. (2019) brings the scientific community a step closer to achieving that goal. For instance, representations derived from protein language models such as the ones generated by the ESM-1b model can identify secondary and tertiary protein structures, offering valuable insights for proteins.

ProteinBERT, for example, is a variant of the Bidirectional Encoder Representations from Transformers (BERT) model specifically tailored for protein sequences. Its aim is to capture global protein representations effectively. The model demonstrates versatility by achieving near state-of-the-art results across numerous protein-related tasks through quick fine-tuning after being trained on UniProtKB/UniRef90 dataset (Boutet et al., 2016; Suzek et al., 2007). ProteinBERT introduces architectural elements specifically designed for proteins, combining language modelling with Gene Ontology (GO) annotation (GO Consortium, 2012) prediction in its pretraining scheme. The architecture of the model enables efficient processing of long sequences and yields impressive performance across multiple benchmarks, including protein structure prediction and post-translational modifications, despite being smaller and faster than competing methods (Brandes et al., 2022). Another significant example of larger protein-based models regards the work (ProtT5 model) of Elnaggar et al. (2021) in which the authors report that these models are able to learn *“some of the grammar of the language of life”*.

Leveraging the theoretical background and the capability of current models to accurately describe protein structures through numerical representations, this potential is exploited to identify patterns within proteins and predict the maximum velocity of the Michaelis–Menten equation as accurately as possible. Instances from the available literature demonstrate that calculations for the kinetic constants of enzymes can rely on specific numerical representations (Kroll et al., 2021; Kroll et al., 2023). Furthermore, it is worth mentioning that the value of *V*_max_ is also related to the concentration of the enzyme in a biological system. By applying the present model, it becomes possible to indirectly estimate the concentration of an enzyme in a biological system.

### 2.7 Introduction of enzymes amino acid proportions

Furthermore, we aimed to delve deeper into matters related to protein structure, specifically investigating how the amino acid composition of an enzyme could impact its physicochemical properties, with a focus on the Michaelis–Menten maximal velocity. Given that another vector was introduced into the dataset which describes the proportions of amino acids that compose an enzyme and has a length equal to 20, similar to the number of amino acids involved in the construction of proteins (Lopez and Mohiuddin, 2020). The introduction of additional features can enrich the dataset and potentially enhance the predictive power of the developing model.

**Fig. 2:**
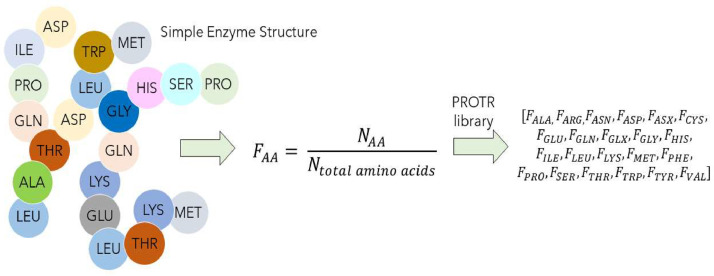
Example of how enzymes amino acid proportions are estimated. Alanine: ALA; Arginine: ARG; Asparagine: ASN; Aspartic acid: ASP; Asparagine or Aspartic acid: ASX; Cysteine: CYS; Glutamic acid: GLU; Glutamine: GLN; Glutamine or Glutamic acid: GLX; Glycine: GLY; Histidine: HIS; Isoleucine: ILE; Leucine: LEU; Lysine: LYS; Methionine: MET; Phenylalanine: PHE; Proline: PRO; Serine: SER; Threonine: THR; Tryptophan: TRP; Tyrosine: TYR; Valine: VAL.

To achieve this, the amino acid sequences collected in previous steps were utilized alongside the PROTR library (Xiao et al., 2015) within the R environment. The PROTR is a comprehensive and powerful R package designed for the analysis of protein sequences and their associated physicochemical properties. With a wide range of functions and classes, PROTR offers a rich set of tools for bioinformatics to extract valuable insights from protein information including the computation of diverse protein descriptors, such as amino acid composition, dipeptide composition, and numerous physicochemical properties. The methodology used to introduce the respective vectors is briefly described in Fig. 2. These enzyme representations will be called PROTR or amino acid proportions from now on.

### 2.8 Introduction of training and test set

In the pursuit of building robust and accurate deep learning models for enzyme kinetics prediction, careful data partitioning is foremost to ensure unbiased evaluation and validation of model performance. In the framework of this study, the dataset was divided randomly, with 70 % of the data allocated for training purposes 10 % for validation and the remaining 20 % reserved for testing. During testing of the optimization algorithm that is described in detail below, it was observed that when 10 % of the data was reserved for validation purposes, the model performed significantly better on unseen data compared to the classic 80/20 ratio. As a result, it was decided to adopt the 70/10/20 ratio for the development of the models. This yielded a training set with 626 entries, a validation set of 90 entries and a test set of 179 entries. It is important to note that only values given in mol/(s*g) were considered in these datasets.

The indication provided by Kroll and Lercher (2023) regarding the presence of similar structures in both the training and test sets underscores the importance of processing the SABIO RK data in a manner that ensures uniqueness of enzyme structures for a given organism, along with the standardization of *V*_max_ units as described in previous sections. Consequently, the present data splitting approach ensures that the model learns from a diverse range of data and is tested on unseen samples, facilitating generalization to new data. It is important to mention that careful measures were taken to ensure that the training, validation, and test datasets contain distinct entries, thus avoiding any instances of data leakage or overlap. This meticulous pre-process approach enhances the reliability and robustness of the model's performance when applied to real-world scenarios and unseen data for the development of in silico NAMs among others. In addition, to further testing the models, they were tested in a set of similar structures that catalyses other metabolic reactions. This dataset consists of 47 entries and resulted from the declined structures from the training set. Last but not least, it should also be mentioned that the distinction between train, validation and test samples prevents the model from overfitting and encourages it to learn underlying patterns in enzyme kinetics given the structural inputs provided.

To ensure consistent and reproducible model training, validation, and testing metrics, the randomly selected training, validation, and test indices were locally stored. These files are also available in the GitHub page. By doing so, we could consistently access the same set of training, validation, and testing data during multiple model training runs. This approach prevents fluctuations in the data distribution across different training sessions, allowing for direct comparisons between the developing models, thus ensuring the consistency and robustness of the results presented in this work.

### 2.9 ANN architecture, hyperparameters optimization, and model fitting

Deep learning has revolutionized predictive modelling, bringing forth powerful and accurate models for a wide array of scientific and industrial applications. In the realm of enzyme kinetics, understanding enzymatic behaviour holds paramount importance for numerous processes such as computational New Approach Methodologies (NAMs), systems biology models, pharmacokinetics, and toxicokinetics. TensorFlow offers high-level APIs that streamline the design, training, validation, and evaluation of deep learning models, making it more accessible and user-friendly for both researchers and practitioners. Keras, an integral part of TensorFlow, provides a simple and intuitive interface, allowing users to swiftly prototype complex neural networks. This simplicity is particularly beneficial in enzyme kinetics research, where domain experts may lack expertise in deep learning methodologies. The user-friendly APIs allow for a focus on the biological aspects of the problem, rather than getting caught up in the technical complexities of deep learning modelling. The versatility of these tools proves invaluable in enzyme kinetics, where the intricate nature of enzymatic behaviour may require different architectural designs to capture specific patterns and interactions. TensorFlow and Keras emerge as significant assets for developing predictive deep learning models, with a particular focus in this study on predicting Michaelis–Menten maximal velocity.

One of the primary challenges in developing deep learning models revolves around optimizing hyperparameters to enhance model reliability and robustness. Traditional methods often involve trial and error to optimize an AI model. However, this approach can be exceedingly time-consuming, especially when dealing with large datasets and models that require extensive training time due to complex architectures. Consequently, the benefits of such approaches compared to experimental methods may diminish. Moreover, even achieving high training metrics such as e.g., *R*^2^ > 0.95 and very low MAE and RMSE does not guarantee finding the optimal combination of hyperparameters despite offering high reliability and performance. Therefore, it is imperative to adopt methodologies that expedite the process of finding the best possible architecture. In this regard, this work adapts the capabilities of KerasTuner (Pon and Krishna Prakash, 2021) to the computing environment of R^1^ through an in-house algorithm to optimize deep learning in order to predict Michaelis–Menten maximal velocity.

KerasTuner is a hyperparameter optimization library for Keras that enables efficient exploration of hyperparameter spaces for complex artificial neural network (ANN) architectures as well as the seamless integration with Keras functionalities. It is widely used in the development of ANN models due to its flexibility and robust search algorithms, including Random Search, Bayesian Optimization, and Hyperband. Here, Random Search was employed. Additionally, KerasTuner was essential for optimizing the model's architecture to ensure accurate prediction of *V*_max_ values while maintaining computational efficiency. It facilitates the automatic exploration of optimal hyperparameter configurations, including learning rates, layer units, and dropout rates, among others. It is important to mention that the use of KerasTuner invariably boosts the performance of neural networks. Last but not least, the library provides a user-friendly and adaptable interface for defining search spaces, selecting search algorithms, and conducting hyperparameter optimization experiments (Pon and Krishna Prakash, 2021).

The models presented in this study are constructed using the sequential API of Keras, a high-level deep learning framework based on TensorFlow. For enzyme kinetic prediction, fully connected neural networks (FCNNs) are employed, and the sequential model offers a convenient approach to building such architectures. These models are constructed and optimized using the functions provided by KerasTuner. During the hyperparameter tuning process, the algorithm is empowered to select key parameters such as the optimizer, learning rate, activation functions, number of neurons per layer, and the inclusion of intermediate layers. The optimization process leverages KerasTuner to systematically explore different hyperparameter combinations to identify the optimal configuration. The algorithm offers a wide range of optimizer choices, including Adadelta (Adaptive Delta), Adam (Adaptive Moment Estimation), Adamax (Adaptive Moment Estimation with Infinity), Nadam (Nesterov Adaptive Moment Estimation), RMSProp (Root Mean Squared Propagation), FTRL (Follow The Regularized Leader), and SGD (Stochastic Gradient Descent). Each optimizer is evaluated with distinct learning rate values, including 1E−05, 1E−04, 1E−03, 1E−02, and 1E−01. The tuning parameters optimized in each case are outlined in the table below (Table 1). The minimum and maximum values denote the upper and lower bounds, while the step indicates the minimum spacing between two subsequent values. The algorithm's selection of hyperparameters significantly influences model performance by balancing underfitting and overfitting. For instance, selecting an overly high learning rate can cause the model to converge too quickly to a suboptimal solution, whereas an excessively low learning rate may lead to slow convergence or getting stuck in a local minimum. Similarly, the number of neurons in each layer and the choice of activation functions impact the model's ability to capture complex patterns in the data. Additionally, the algorithm is capable of selecting from a plethora of built-in loss functions, including Mean Absolute Error (MAE), Mean Squared Error (MSE), Mean Squared Logarithmic Error, and Log-Cosh, to determine the most reliable model. The only metric function utilized is *R-squared*, which is also the metric used by the algorithm to choose the best model. Since Keras does not offer *R-squared* as a built-in metric, an in-house function was implemented for its estimation. This function is also considered as a potential loss function option during tuning. A reliable architecture for an artificial neural network also involves activation functions and multiple layers. Activation functions are significantly important in introducing non-linearity to the deep learning model, allowing it to learn complex patterns from the input data. The algorithm is equipped with the option to select from multiple activation functions, including ReLU (Rectified Linear Unit), ELU (Exponential Linear Unit), SELU (Scaled Exponential Linear Unit), Hard Sigmoid, Linear, Sigmoid, SoftMax, Tanh, Exponential, GELU (Gaussian Error Linear Unit), and Swish, with their default arguments as provided by the Keras library. Furthermore, the algorithm allows for the selection of seven dense layers and two dropout layers: a normal one and a Gaussian. The first and last dense layers must be chosen, and each dense layer can have a different activation function. All layers containing neurons have the option to select from 1 to 2048 neurons, except for the last layer, where the algorithm necessarily selects from 1 to 4 neurons, and the activation function is set to Linear by default. During development, models consistently performed better when the last layer had a Linear activation function (data not shown). As a result, the Linear activation function was deemed appropriate for regression tasks where the output needs to be a continuous value. The algorithm also determines whether to include the intermediate five layers, all of which are equipped with the previously mentioned activation functions and neuron options.

**Table 1:** Parameters were finely tuned using Keras Tuner functions. In cases where no tuning parameters were provided, the algorithm defaults to using preset values for each optimizer.

Optimizer	Tuning parameter	Minimum value	Maximum value	Step
*Adadelta (Adaptive Delta)*	RHO	0.01	0.99	0.01
*Adam (Adaptive Moment Estimation)*	BETA 1	0.01	0.99	0.01
	BETA 2	0.001	0.999	0.001
*Adamax (Adaptive Moment Estimation with Infinity)*	BETA 1	0.01	0.99	0.01
	BETA 2	0.001	0.999	0.001
*Nadam (Nesterov Adaptive Moment Estimation)*	BETA 1	0.01	0.99	0.01
	BETA 2	0.001	0.999	0.001
*RMSProp (Root Mean Squared Propagation)*	RHO	0.01	0.99	0.01
*FTRL (Follow The Regularized Leader)*	Power of learning rate	–	−0.01	0.01
*SGD (Stochastic Gradient Descent)*				

To mitigate overfitting, which occurs when the model memorizes the training data rather than learning general patterns, dropout layers were introduced into the Keras Tuner algorithm. Dropout randomly deactivates neurons during training, compelling the network to learn more robust and generalized representations, thereby enhancing its generalization ability. Additionally, dropout serves as a regularization technique, making the model less sensitive to noise. In addition to the option of including dropout layers in the network architecture, the algorithm was also provided the flexibility to specify the dropout fraction, ranging between 0.01 and 0.99 with a step of 0.01 in both Gaussian and the regular dropout layers.

Finally, the main criteria for the adoption of a model in each case concerned metrics such as a high *R-squared* in the test dataset (and unseen data). The detailed results obtained in each use-case as well as the deep learning models developed are presented in detail in the next chapter. It is worth mentioning that the number of neurons in each layer gradually decreases until the final layer. The progressive reduction can be attributed to the ability of the model to extract increasingly abstract and meaningful features from the data as it delves deeper into the network.

### 2.10 Statistical analysis plan to validate performance of the developing models

To enhance the credibility of the results presented in this scientific work, it is essential to compare the predictions generated by the deep learning models in the following sections with experimentally recorded values applying widely accepted statistical methods. To achieve this, two statistical methods have been employed. In the first approach, linear regression is utilized to compare the predictions with the experimental data. Linear regression is commonly employed to assess the performance of predictive models by analysing their predictions against experimental measurements. This method offers a straightforward yet robust means to establish the relationship between two continuous variables-the model outputs and the corresponding experimental observations. Similar methodologies have also been reported in the literature such as the work of Choetkiertikul et al. (2018).

The second method employed to enhance the reliability of the predictions generated by the models presented below involves calculating the Pearson correlation coefficient (r). Pearson's coefficient serves as a robust metric for quantifying the strength and direction of the linear relationship between two variables. A value close to 1 indicates a strong positive linear relationship, while values approaching 0 suggest little to no linear correlation. Importantly, Pearson's coefficient is resilient to outliers, which is advantageous when dealing with experimental data that may contain noise or outliers, such as datasets with multiple variations in *V*_max_ values. Its adoption in comparing deep learnint predictions with experimental measurements provides a universally accepted measure of association. Pearson correlation coefficient (r) is a widely adopted metric to assess the performance of a deep learning model against experimentally measured values and has been also broadly reported in the literature as well (Preuer et al., 2018).

Finally, in assessing the performance of deep learning models, the inclusion of metrics such as *R-squared* (coefficient of determination), Mean Absolute Error (MAE), and Root Mean Squared Error (RMSE) is imperative for a comprehensive evaluation. *R-squared* provides valuable insight into the proportion of variance in the dependent variable that is predictable from the independent variables, thereby offering a measure of model adequacy. MAE, characterized by its simplicity and interpretability, offers a robust indication of the average magnitude of errors present in predictions, facilitating a clear understanding of model accuracy. RMSE, akin to MAE but incorporating the square root of the average squared differences between predictions and actual values, tends to penalize larger errors more significantly, thus providing a nuanced perspective on model performance. The combined utilization of these metrics provides a comprehensive evaluation, allowing for a thorough assessment of both the predictive capability and the precision of deep learning models that will be presented in this study.

## 3 Results and discussion

### 3.1 Deep learning model based on reaction fingerprints

The *V*_max_ value of an endogenous metabolic reaction is strongly associated with both the structure of the enzyme and the chemical transformation involved. The integration of the metabolites structure into the deep learning models carried out with the employment of molecular fingerprints as detailed previously. Due to this consideration, four different molecular fingerprints were evaluated, which included RCDK standard fingerprints (1024 bits), MACCS keys (166 bits), PubChem fingerprints (881 bits), and E-States fingerprints (79 bits). These fingerprints were utilized to construct reaction fingerprints and were compared in terms of their performance to predict Michaelis–Menten maximal velocity. The construction of the reaction fingerprints relies on the difference between the aggregated fingerprints of each substrate and product resulting in a single vector with size equal to the initial fingerprint (Fig. 1).

**Fig. 3:**
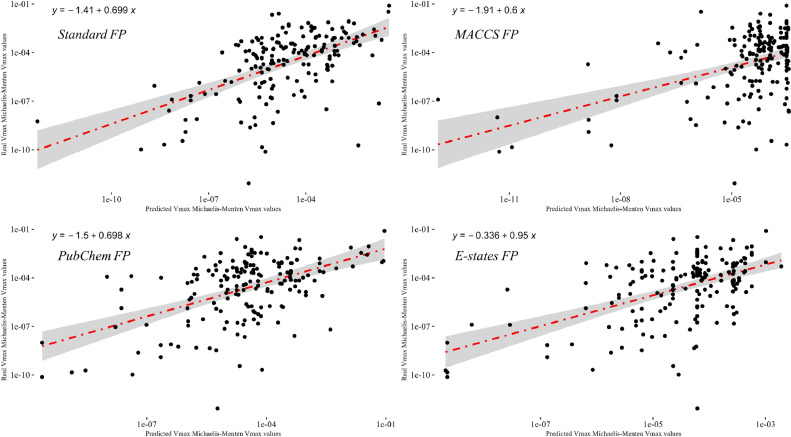
Comparison of experimentally measured Michaelis–Menten *V*_max_ with those predicted for the different reaction fingerprints. Upper left RCDK standard fingerprints (1024 bits); Upper right MACCS keys (166 bits); Bottom left PubChem fingerprints (881 bits); Bottom right E-States fingerprints (79 bits). All *V*_max_ values are given at mol/(s*g).

In Table 2, a brief summary of how the deep learning models performed on the test set is presented, while in Fig. 3, the comparison of experimentally measured Michaelis–Menten *V*_max_ values with those predicted for all the reaction fingerprints is illustrated. It is worth noting that the models themselves are not provided on the GitHub page.

**Table 2:** Test metrics and statistical presentation of the model trained only on reaction fingerprints.

Reaction fingerprint	*R*^2^	MAE	RMSE	Pearson coefficient
*RCDK standard (1024 bits)*	0.32	0.63	0.85	0.56
*MACCS keys (166 bits)*	0.27	0.66	0.89	0.51
*PubChem (881 bits)*	0.28	0.69	0.87	0.52
*E-States (79 bits)*	0.29	0.64	0.84	0.53

* *R*^2^: *R* squared; MAE: Mean Absolute Error; RMSE: Root Mean Squared Error; Pearson Correlation Coefficient

None of the models developed at this stage produced reliable results, indicating that neither molecular nor reaction fingerprints alone can robustly predict the Michaelis–Menten *V*_max_ of an enzymatic transformation. Specifically, four fully connected artificial neural networks were built and fine-tuned using the Keras Tuner architecture and the methodology described previously.

It is evident that most of the fingerprints exhibit similar metrics, with the standard fingerprint generally outperforming the others as shown in Table 2 and Fig. 3. Specifically, the model trained exclusively the RCDK standard reaction fingerprint achieved a coefficient of determination of 0.32, a MAE of 0.63, an RMSE of 0.85, and a Pearson coefficient of 0.56 between the predictions and the measured values (test set). On the other hand, the models based on the other reaction fingerprints exhibited relatively similar metrics compared to the model trained on the RCDK fingerprint. Specifically, the model based on MACCS keys achieved an *R*^2^ of 0.27 while the PubChem and E-States achieved values of 0.28 and 0.29 respectively. From the overall analysis of the models at this stage of development, it is unclear whether more bits lead to more consistent predictions or if additional information regarding the chemical structure is incorporated into the neural network. The reactions in the datasets were not unique because they resulted from different enzyme-catalysed reactions.

### 3.2 Deep learning model based on PROTR amino acid proportions

In the quest to explore potential connections between amino acid proportions and Michaelis–Menten maximal velocity, a fully connected neural network (FCNN) model was specifically constructed only employing enzyme amino acid proportions given the optimization procedure described in detail previously. The objective was to unravel potential relationships between enzyme amino acid compositions and enzyme kinetics. However, despite testing multiple model architectures, the performance of the FCNN model did not yield promising results. A very low *R*-squared (*R*^2^ < 0.1) and Pearson coefficient (*r* ≈ 0) indicated weak correlations between the input features and Michaelis–Menten *V*_max_. Consequently, the performance of the FCNN model was not reliable. A summary of the model presented here is depicted in Table 3. The model is not provided in the GitHub page.

**Table 3:** Test metrics and statistical presentation of the model trained only on amino acid proportions.

Amino acid proportions (PROTR)	*R*^2^	MAE	RMSE	Pearson coefficient
	<0.1	0.76	1.06	≈0

* *R*^2^: *R* squared; MAE: Mean Absolute Error; RMSE: Root Mean Squared Error; Pearson Correlation Coefficient

Multiple model architectures were tested in an attempt to identify the most suitable configuration for establishing correlations between amino acid proportions (PROTR) and the *V*_max_ values. Through experimentation with the in-house Keras Tuner algorithm, models with numerous hidden layers, activation functions, and regularization techniques were explored. Despite the comprehensive exploration, the performance of the model remained unsatisfactory. Nevertheless, the pursuit of uncovering potential connections between AA proportions and enzyme kinetics remains a significant research endeavour, necessitating the inclusion of more data points and a more comprehensive dataset.

It is important to emphasize that there may indeed be common amino acid ratios in enzymes and proteins, given that the number of amino acids used to construct such structures is finite. However, it is also a fact that different ratios of amino acids can result in different protein structures, sequences, and spatial arrangements, which may not be effectively captured by the fully connected neural network (FCNN) model. As a result, there is a discrete number of combinations of ratios that can be explored, but they may not adequately describe all kinetic values. Therefore, the tests conducted confirm that relying solely on the ratio of amino acids to proteins as a predictor of Michaelis–Menten maximal velocity is not trustworthy. This investigation underscores the importance of continued research and innovation in exploring the relationships between enzyme kinetics and enzymatic characteristics. Last but not least, considering the highly unreliable performance of the current model, it is not further tested on similar amino acid proportions.

### 3.3 Deep learning model based on reaction fingerprints and PROTR proportions

**Fig. 4:**
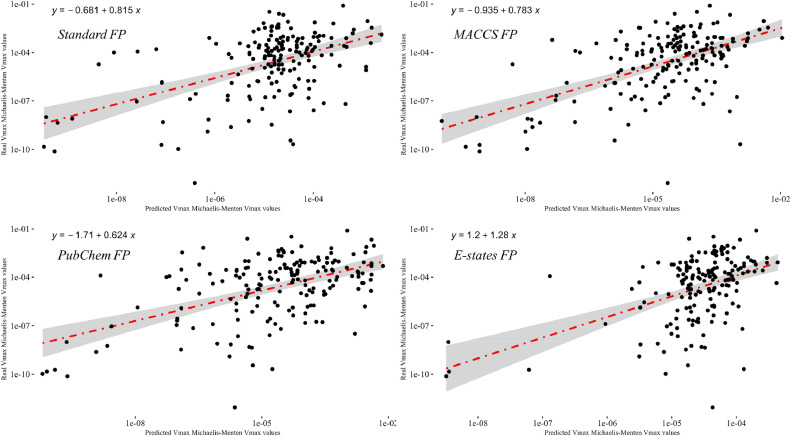
Comparison of experimentally measured Michaelis–Menten *V*_max_ with predicted values for combined reaction fingerprints and amino acid proportions. Upper left RCDK standard fingerprints (1024 bits); Upper right MACCS keys (166 bits); Bottom left PubChem fingerprints (881 bits); Bottom right E-States fingerprints (79 bits). All *V*_max_ values are given at mol/(s*g).

In this endeavour, reaction fingerprints were integrated with amino acid proportions, which offer valuable information about enzyme composition. The goal was to leverage the combined features to establish connections with the Michaelis–Menten maximal velocity, potentially unveiling deeper insights into enzyme kinetics. Due to that fact four different molecular fingerprints were assessed, which included RCDK standard fingerprints (1024 bits), MACCS keys (166 bits), PubChem fingerprints (881 bits), and E-States fingerprints (79 bits). The summary results of the performance of all developed models are presented in Table 4. Comparisons between experimentally measured *V*_max_ values and predictions are depicted in Fig. 4. The models are not provided on the GitHub page.

**Table 4:** Test metrics and statistical presentation of the model trained on AA proportions and reaction fingerprints.

PROTR and reaction fingerprints	*R*^2^	MAE	RMSE	Pearson coefficient
*PROTR and RCDK standard (1024 bits)*	0.25	0.68	0.87	0.50
*PROTR and MACCS keys (166 bits)*	0.27	0.66	0.83	0.57
*PROTR and PubChem (881 bits)*	0.27	0.71	0.90	0.52
*PROTR and E-States (79 bits)*	0.23	0.67	0.88	0.47

* *R*^2^: *R* squared; MAE: Mean Absolute Error; RMSE: Root Mean Squared Error; Pearson Correlation Coefficient

As previously mentioned, amino acid proportions can have a finite number of combinations and may not offer clear information about protein structure. However, it became evident that they could not provide robust estimates of the *V*_max_ values when they employed solely. At this point, the idea was to combine them with the reaction fingerprints and assess to what extent they could improve the existing metrics.

To train and optimize the models, the methodology described in detail previously was employed. The architectures based on MACCS keys and PubChem performed better compared to RCDK fingerprint and ESTATES, recording *R-squared* values of 0.27. In contrast, the model based on the RCDK fingerprint showed an *R-squared* of 0.25, while the one trained on ESTATES showed an *R-squared* of 0.23. Furthermore, the model based on MACCS keys exhibited the lowest MAE, which was equal to 0.66, comparable to the other models. Even at this stage, it is not evident that the number of bits provided by a fingerprint provides the FCNN with a greater amount of information about the Michaelis–Menten maximal velocity, as no clear pattern emerges to support this claim. Lastly, it should be noted that the inclusion of amino acid proportions did not improve the performance of the existing models. Finally, due to the consistently unreliable performance of the existing models, they are not subjected to further testing on similar amino acid structures.

### 3.4 Deep learning model based on ESM-1b representations

The next step of the presented approach involves incorporating ESM-1b representations as the basis to train and optimize the fully connected neural network. Utilizing numerical protein representations derived from amino acid sequences has proven to be successful in deep learning architectures (Brandes et al., 2022; Elnaggar et al., 2021). Originally designed for NLP tasks such as token or sentence classification, these architectures have been adapted for enzyme sequences, treating amino acid sequences as words (Alley et al., 2019; Brandes et al., 2022; Rives et al., 2021). ESM-1b representations, developed by the Facebook AI Research team, rely on transformer architectures, which represent a cutting-edge approach in developing NLP models (Rives et al., 2021). Last but not least, it should be noted that resulting model representations have been found to contain very valuable information regarding the enzyme structure (Goldman et al., 2022; Kroll et al., 2022b; Rives et al., 2021) as well as to effectively summarize the evolutionary relationships and sequence-structure alignments of enzymes.

**Fig. 5:**
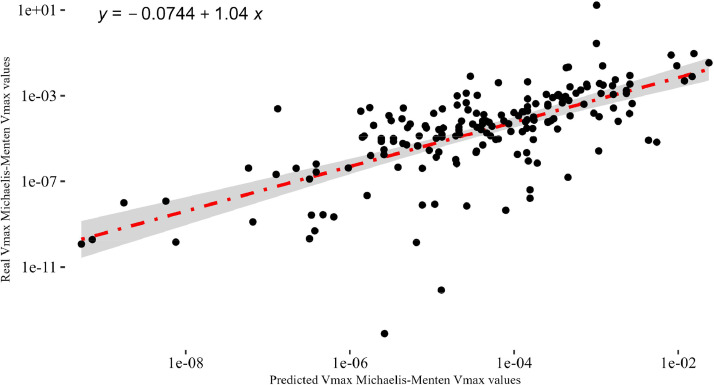
Comparison of experimentally measured Michaelis–Menten *V*_max_ with predictions from the model utilizing only enzyme representations. All *V*_max_ values are given in mol/(s*g). The structures used for the experimentally measured values in the test set have not been encountered previously by the model.

The combination of ESM-1b representations with conventional enzyme kinetic features introduces multi-modal learning capabilities to the FCNN model. This enriched architecture enables the model to jointly learn from diverse sources of information, effectively capturing both the kinetic and structural characteristics of enzymes. In addition, incorporating ESM-1b representations in the FCNN training process enables information fusion. The model learns to effectively combine the structural information from ESM-1b representations with the kinetic features, potentially leading to more robust and accurate predictions. This is something corroborated by the performance of the model on the test set as summarized in Table 5. The comparison between the data derived from SABIO RK and the predictions made by the model is depicted in Fig. 5. Additionally, the model presented here is available on the GitHub page.

**Table 5:** Metrics for unknown structures (test set) and statistical presentation of the model trained exclusively on enzyme representations.

ESM-1b representations	*R*^2^	MAE	RMSE	Pearson coefficient
	0.45	0.51	0.79	0.67

* *R*^2^: *R* squared; MAE: Mean Absolute Error; RMSE: Root Mean Squared Error; Pearson Correlation Coefficient

Michaelis–Menten *V*_max_ is strongly influenced by the structure of the enzyme, playing a fundamental role in enzyme-catalysed transformations. The ESM-1b representations result in a 1280-dimensional array that includes valuable information about the enzyme structure. Given this, model training and optimization were conducted using the Keras Tuner framework, as detailed previously. The model presented here is capable of predicting *V*_max_ values using the ESM-1b representations. On unseen data, this model achieved an *R*^2^ of 0.45, a MAE of 0.51, and a robust Pearson coefficient of 0.67.

**Fig. 6:**
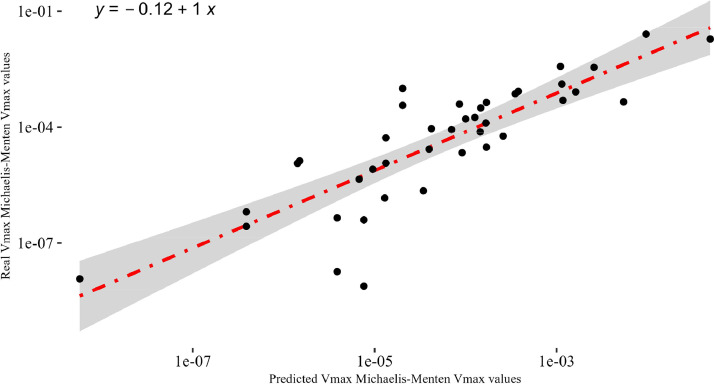
Comparison of experimentally measured Michaelis–Menten *V*_max_ with predictions from the model utilizing only enzyme representations. All *V*_max_ values are given in mol/(s*g). The structures were previously encountered by the model and were included in the training set, but they catalyze other reactions.

During the data splitting procedure, an additional dataset was introduced to test the model developed here. This dataset exclusively included enzyme structures from the training set that the model had previously encountered. Structures from the test and validation sets were not included in this dataset. This allowed for assessing the model's performance on a set of structures it was familiar with but had not seen in combination with the specific reaction fingerprint and *V*_max_ value. A summary of the model's performance on this dataset is illustrated in Table 6, while the comparison of predictions with the experimentally measured *V*_max_ values is depicted in Fig. 6. As anticipated, the model exhibited improved performance on this dataset compared to the previous one, achieving a coefficient of determination of 0.70, a mean absolute error of 0.31, and an outstanding Pearson coefficient of 0.84. These results strengthen the idea of a strong association between enzyme structures and Michaelis–Menten *V*_max_.

**Table 6:** Metrics for similar structures and statistical presentation of the model trained exclusively on enzyme representations. The structures were previously encountered by the model and were included in the training set, but they catalyze other reactions.

ESM-1b representations	*R*^2^	MAE	RMSE	Pearson coefficient
	0.70	0.31	0.43	0.84

* *R*^2^: *R* squared; MAE: Mean Absolute Error; RMSE: Root Mean Squared Error; Pearson Correlation Coefficient

### 3.5 Deep learning model based on ESM-1b and PROTR amino acid proportions

**Fig. 7:**
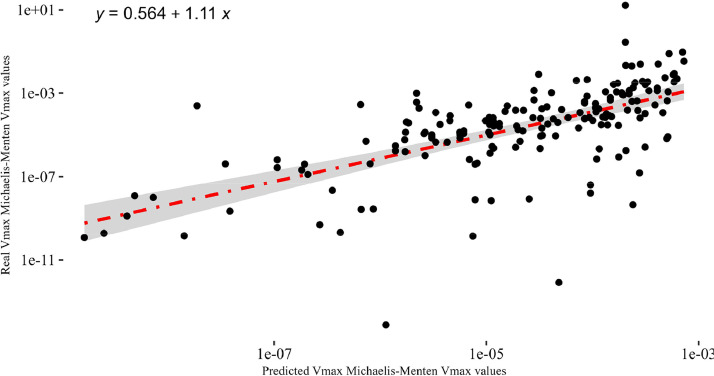
Comparison of experimentally measured Michaelis–Menten *V*_max_ with predictions from a model utilizing enzyme representations and amino acid proportions. All *V*_max_ values are given in mol/(s*g). The structures used for the experimentally measured values in the test set have not been encountered previously by the model.

In the quest to empower the connections between ESM-1b representations, amino acid proportions were also included in the dataset. The integration of amino acid proportions alongside ESM-1b representations aimed to leverage the prediction capabilities of the model, as it was expected to capture more comprehensive insights regarding enzymatic behaviour. It is worth mentioning that the amino acid composition of an enzyme, which is a piece of information concerning the enzyme structure, does not improve the performance of the model. The performance of the model on the test set is summarized on Table 7 while the comparison of the experimentally measured data with the predictions is illustrated on Fig. 7. This model is not available on the GitHub page.

**Table 7:** Metrics for unknown structures (test set) and statistical presentation of the model trained solely on enzyme representations and amino acid proportions.

ESM-1b representations & amino acid proportions (PROTR)	*R*^2^	MAE	RMSE	Pearson coefficient
	0.40	0.55	0.83	0.63

* *R*^2^: *R* squared; MAE: Mean Absolute Error; RMSE: Root Mean Squared Error; Pearson Correlation Coefficient

The architecture for developing the FCNN model presented here followed the detailed description provided previously. The objective was to enhance the potential relationships between enzyme compositions and structure with the Michaelis–Menten maximal velocity. However, the resulting model did not outperform the one trained solely on the ESM-1b representations when evaluated on the test set. The resulting metrics were an *R-squared* of 0.40 and an MAE of 0.55. The inclusion of PROTR in the predictors did not lead to improvement in the model's performance on the unseen data.

**Fig. 8:**
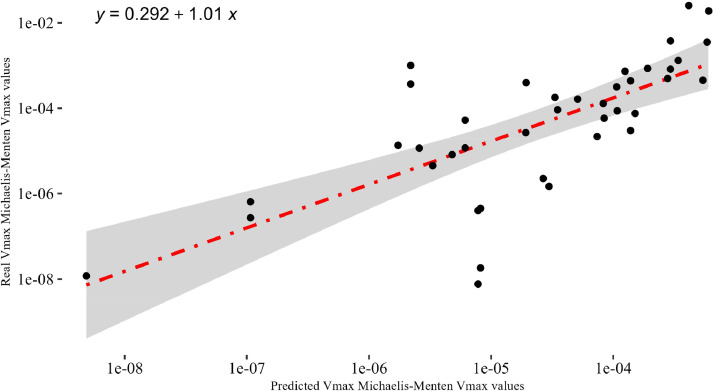
Comparison of experimentally measured Michaelis–Menten *V*_max_ with predictions from the model utilizing enzyme representations and amino acid proportions. All *V*_max_ values are given in mol/(s*g). The structures were previously encountered by the model and were included in the training set, but they catalyze other reactions.

To further assess the performance of the current model, it was applied to enzyme structures identified during the data splitting procedure. This data has encountered before by the model during training and contained similar structures with the difference that they catalyse different metabolic reactions, thus different reaction fingerprints. A summary of the results is provided in Table 8, and the comparison between the data from SABIO RK and the predictions of the present model on this set is presented in Fig. 8. As expected, the model's performance on similar structures appeared to be improved, with a relatively higher *R-squared* value of 0.52 and a significantly improved Pearson coefficient of 0.72. However, the stance remains that PROTR proportions cannot serve as a reliable predictor for Michaelis–Menten *V*_max_, as even in this case they did not enhance the model's performance.

**Table 8:** Metrics on similar structures and statistical presentation of the model trained on enzyme representations and amino acid proportions. The structures were previously encountered by the model and were included in the training set, but they catalyze other reactions.

ESM-1b representations & amino acid proportions (PROTR)	*R*^2^	MAE	RMSE	Pearson coefficient
	0.52	0.42	0.56	0.72

* *R*^2^: *R* squared; MAE: Mean Absolute Error; RMSE: Root Mean Squared Error; Pearson Correlation Coefficient

### 3.6 Deep learning model based on ESM-1b representations and reaction fingerprints

Some of the deep learning models described and trained in the previous paragraphs have demonstrated excellent performance on the test dataset, yielding high metrics and showcasing its efficacy in enzyme kinetics prediction, specifically the ESM-1b based model. However, it is essential to address a critical challenge posed by multi-functional enzymes, where a single enzyme can catalyse more than one biochemical reaction, leading to diverse kinetic profiles and intricate interactions within biological systems. This scenario introduces complexities that may increase uncertainty levels in the predicted data, particularly in the context of developing mechanistic systems biology models.

**Fig. 9:**
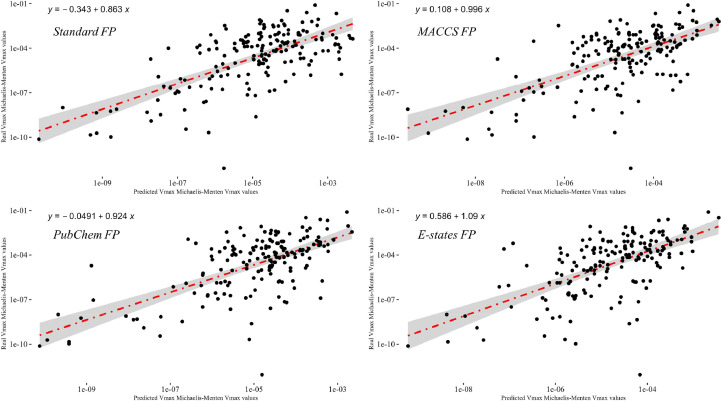
Comparison of experimentally measured Michaelis–Menten *V*_max_ with predictions for all reaction fingerprints combined with ESM-1b representations. Upper left: RCDK standard fingerprints (1024 bits); Upper right: MACCS keys (166 bits); Bottom left: PubChem fingerprints (881 bits); Bottom right: E-States fingerprints (79 bits). All *V*_max_ values are given in mol/(s*g).

To overcome this issue, for each chemical reaction, a reaction fingerprint was estimated to depict the compounds participant in a metabolic transformation. Consequently, in the training data, each entry includes the ESM-1b representations and the reaction fingerprints. This is something that slightly improved the performance of the developed model considering not only the structure of the enzyme but also the reaction it catalyses. In fact, the estimation of the reaction fingerprint allows for the approximation of the *V*_max_ of bidirectional reactions as well. Training procedure described in detail previously. The performance of the models on the unseen data is provided in Table 9 while the comparison of the experimentally measured data with the predictions is given in Fig. 9. The model trained on ESM-1b representations and the reaction fingerprints of RCDK is provided on GitHub page.

**Table 9:** Metrics on the unknown structures (test set) and statistical presentation of the model trained on the enzyme representations and reaction fingerprints.

ESM-1b and reaction fingerprints	*R*^2^	MAE	RMSE	Pearson coefficient
ESM-1b *and RCDK standard (1024 bits)*	0.46	0.60	0.75	0.67
ESM-1b *and MACCS keys (166 bits)*	0.40	0.58	0.77	0.63
ESM-1b *and PubChem (881 bits)*	0.43	0.57	0.77	0.65
ESM-1b *and E-States (79 bits)*	0.39	0.58	0.78	0.62

* *R*^2^: *R* squared; MAE: Mean Absolute Error; RMSE: Root Mean Squared Error; Pearson Correlation Coefficient

The metrics achieved using the ESM-1b representations and the RCDK estimated standard reaction fingerprints on the unseen data were an *R*^2^ equal to 0.46 and an MAE equal to 0.60. The Pearson coefficient (r) is 0.67. On the other hand, when combining the enzyme representations with the PubChem reaction fingerprints, the model achieved an *R*^2^ value of 0.43 and an r of 0.65, which are very close to the performance of the model trained on RCDK fingerprints. However, the models trained on MACCS, and E-states fingerprints showed slightly lower performance, with *R*^2^ values of 0.40 and 0.39, respectively. The MAE indices were comparable for both models, with a value of 0.58, while the Pearson coefficient for the model trained on MACCS was 0.63 and 0.62 for the one trained on E-states fingerprints.

**Fig. 10:**
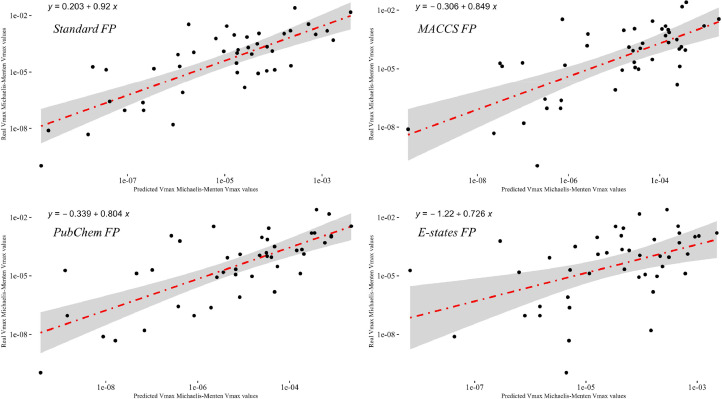
Comparison of experimentally measured Michaelis–Menten *V*_max_ with predictions for all reaction fingerprints combined with ESM-1b representations. Upper left: RCDK standard fingerprints (1024 bits); Upper right: MACCS keys (166 bits); Bottom left: PubChem fingerprints (881 bits); Bottom right: E-States fingerprints (79 bits). All *V*_max_ values are given in mol/(s*g). The structures were previously encountered by the model and were included in the training set. The reactions were not previously met by the model.

Furthermore, the models presented here were also tested on known structures that catalyse different reactions, as conducted previously. The resulting performance metrics are portrayed in Table 10, and the comparisons between predictions and experimentally measured values are shown in Fig. 10.

**Table 10:** Metrics for similar structures and statistical presentation of the model trained on enzyme representations and reaction fingerprints. The structures were previously encountered by the model and were included in the training set. The reactions were not previously met by the model.

ESM-1b and reaction fingerprints	*R*^2^	MAE	RMSE	Pearson coefficient
ESM-1b *and RCDK standard (1024 bits)*	0.62	0.51	0.65	0.78
ESM-1b *and MACCS keys (166 bits)*	0.49	0.54	0.70	0.70
ESM-1b *and PubChem (881 bits)*	0.54	0.52	0.72	0.74
ESM-1b *and E-States (79 bits)*	0.43	0.58	0.79	0.68

* *R*^2^: *R* squared; MAE: Mean Absolute Error; RMSE: Root Mean Squared Error; Pearson Correlation Coefficient

As expected, all models demonstrated significantly enhanced performances when evaluated on known structures compared to their performances on unknown ones. Specifically, the model trained on the RCDK fingerprints achieved an *R*^2^ of 0.62 and a Pearson coefficient of 0.78. Similarly, the model based on PubChem fingerprints achieved an *R-squared* value of 0.54, a mean absolute error of 0.52, and a Pearson coefficient of 0.74. However, the other two models performed less satisfactorily, with *R-squared* values below 0.5 and Pearson coefficients close to 0.70.

It is essential to highlight that incorporating reaction fingerprints does not enhance the model's performance compared to solely utilizing the ESM-1b representations. Moreover, it appears that providing more bits to the model, the better the performances recorded due to the fact that more pieces of information regarding the molecular structures are available during the training process. This is something that is confirmed only at this stage and in any case, more should be performed by considering more fingerprints with higher variations. The disparity observed in known structures between the model trained exclusively on enzyme representations and the one trained on both enzyme representations and standard fingerprints is likely due to the model encountering similar structures with unknown reactions. Consequently, the model exhibits lower performance; however, its significance for applications persists.

### 3.7 Deep learning model based on ESM-1b, AA proportions and reaction fingerprints

**Fig. 11:**
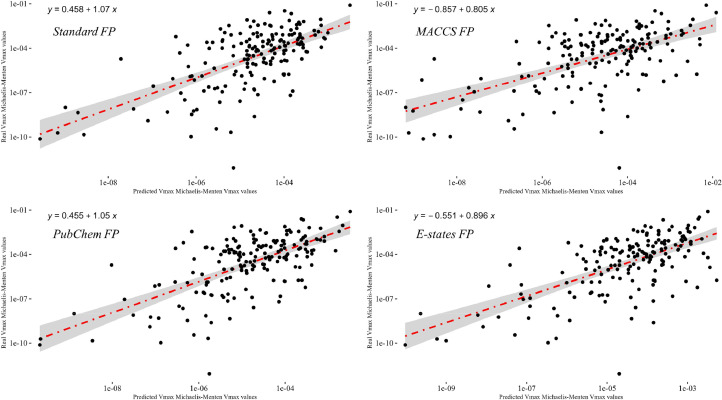
Comparison of experimentally measured Michaelis–Menten *V*_max_ with predictions for all reaction fingerprints combined with ESM-1b representations and amino acid proportions. Upper left: RCDK standard fingerprints (1024 bits); Upper right: MACCS keys (166 bits); Bottom left: PubChem fingerprints (881 bits); Bottom right: E-States fingerprints (79 bits). All *V*_max_ values are given in mol/(s*g).

Finally, four additional models were trained, incorporating ESM-1b representations, reaction molecular fingerprints, and PROTR amino acid proportions as attributes. These models were constructed using the optimization procedure of the in-house methodological pipeline. A summary of the resulting metrics on the test set (unseen data) is presented in Table 11, while the comparison between the data sourced from SABIO RK and the predictions is illustrated in Fig. 11. These models are not available in the GitHub page.

**Table 11:** Metrics for the unknown structures (test set) and statistical presentation of the models trained in enzyme representations, PROTR amino acid proportions, and reaction fingerprints.

ESM-1b, PROTR and reaction fingerprints	*R*^2^	MAE	RMSE	Pearson coefficient
ESM-1b, PROTR *and RCDK standard (1024 bits)*	0.41	0.58	0.77	0.63
ESM-1b, PROTR *and MACCS keys (166 bits)*	0.36	0.60	0.81	0.59
ESM-1b, PROTR *and PubChem (881 bits)*	0.44	0.58	0.75	0.66
ESM-1b, PROTR *and E-States (79 bits)*	0.42	0.56	0.76	0.64

* *R*^2^: *R* squared; MAE: Mean Absolute Error; RMSE: Root Mean Squared Error; Pearson Correlation Coefficient

In this scenario, the performance metrics of the trained models exhibit variability, ranging from an *R-squared* of 0.36 for MACCS to 0.44 for PubChem fingerprints. However, the models demonstrate similar outcomes concerning MAE, RMSE, and Pearson coefficients, with values closely aligned across all models. Moreover, it appears that integrating amino acid ratios into the neural network does not enhance models’ performance, thus rendering them unreliable predictors for Michaelis–Menten *V*_max_.

**Fig. 12:**
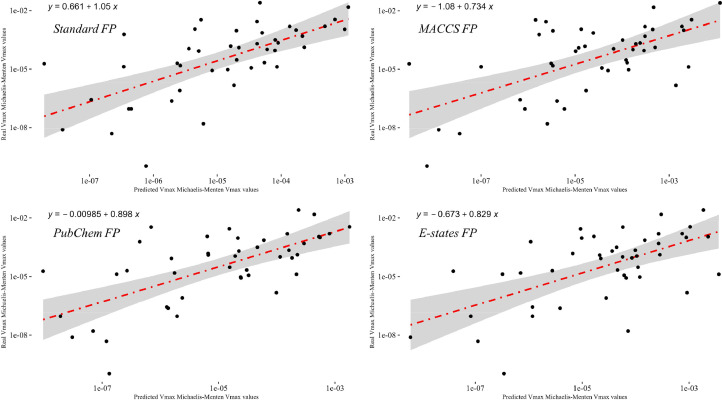
Comparison of experimentally measured Michaelis–Menten *V*_max_ with predictions for all reaction fingerprints combined with ESM-1b representations and amino acid proportions. Upper left: RCDK standard fingerprints (1024 bits); Upper right: MACCS keys (166 bits); Bottom left: PubChem fingerprints (881 bits); Bottom right: E-States fingerprints (79 bits). All *V*_max_ values are given in mol/(s*g). The structures were previously encountered by the model, they were included in the training set, but they catalyze other reactions.

The performance of the model on known structures is showcased in Table 12. Additionally, the experimentally measured values are compared with the model predictions are depicted in Fig. 12.

**Table 12:** Metrics for similar structures and statistical presentation of the model trained on enzyme representations, amino acid proportions, and reaction fingerprints. The structures were previously encountered by the model and were included in the training set, but they catalyze other reactions.

ESM-1b, PROTR and reaction fingerprints	*R*^2^	MAE	RMSE	Pearson coefficient
ESM-1b, PROTR *and RCDK standard (1024 bits)*	0.44	0.54	0.71	0.69
ESM-1b, PROTR *and MACCS keys (166 bits)*	0.39	0.58	0.76	0.61
ESM-1b, PROTR *and PubChem (881 bits)*	0.46	0.55	0.72	0.69
ESM-1b, PROTR *and E-States (79 bits)*	0.45	0.56	0.73	0.68

* *R*^2^: *R* squared; MAE: Mean Absolute Error; RMSE: Root Mean Squared Error; Pearson Correlation Coefficient

At this juncture, the models demonstrate significantly enhanced performance when tested on known structures compared to unknown ones. The model trained on PubChem Fingerprints exhibited the best performance in this scenario, with an MAE of 0.58, an *R-squared* of 0.46, and a Pearson coefficient of 0.69. A similar Pearson coefficient was observed for the model trained on RCDK fingerprints. Notably, the performances of all models were relatively close to each other, except for the model based on MACCS fingerprints. Overall, the inclusion of amino acid proportions did not serve as a reliable predictor for *V*_max_ estimation, as their combination with existing structures did not enhance the performance of the models. Finally, the number of bits provided by each fingerprint does not follow a consistent pattern, i.e., more bits lead to more pieces of information into the artificial neural network.

### 3.8 Comparison with the models in the literature

The prediction of Michaelis–Menten maximal velocity is somewhat controversial due to its dependence not only on enzyme structure but also on enzyme concentration within a cellular system which does not follow a steady state format. Consequently, it is not extensively explored in the literature as its determination can be performed indirectly. Reliable models already exist for determining turnover numbers (*k*_cat_) and enzyme concentrations in in vitro systems, making it less complicated. Therefore, there are currently no comparable models available to directly assess the performance of the models described in this paper. However, we will indirectly compare them with models that calculate *k*_cat_ and Michaelis–Menten constants (*K*_m_), assuming a proportional relationship between these quantities.

Kroll et al. (2023) introduced the TurNuP model, which follows a methodology similar to the one presented here. Their deep learning approach utilizes a transformer network to characterize enzyme structure as well as reaction fingerprints to illustrate molecular structures into the neural network. They constructed multiple models to conclude in the most efficient one for predicting turnover numbers for enzymatic reactions sourced from BRENDA database (Jeske et al., 2019) and SABIO RK (Wittig et al., 2012). They reported coefficients of determination for the model trained solely on reaction fingerprints, reaching 0.38, which is close to our achieved results. Their approach to estimating reaction fingerprints is similar to ours, although they used molecular fingerprints with a higher number of bits. Additionally, when they trained their model solely on enzyme representations, they achieved a coefficient of determination of 0.37 for unseen data and 0.67 for known structures. It is important to note that both studies utilized the model developed by Rives et al. (2021) for the enzyme representations. In our case, our model achieved an *R-squared* of 0.45 for unseen data and 0.70 for known structures. Last but not least, the authors introduced a combined model with enzyme representations and reaction fingerprints, aligning with the achievements of the model present in this scientific work. Specifically, our RCDK- and ESM-1b-based model showed similar Pearson coefficients with their deep learning model.

Heckmann et al. (2018) devised a machine learning model aimed at predicting turnover numbers for metabolic reactions in *Escherichia coli*. Their dataset was constrained, consisting of only 215 entries and it was species specific. Predictors utilized in their model included parameters such as the enzyme's active site, experimental temperature, and pH. Their model achieved a coefficient of determination of 0.34 on the test set, a value similar to the coefficient we attained during our training procedures.

Another model found in literature focusing on characterizing the kinetic parameters of enzymatic reactions concerns the work of Li et al. (2022). They developed a model to determine turnover numbers, utilizing information from the amino acid sequence for only one of the substrates involved in the reaction. One notable difference in their approach to encoding enzyme structures compared to ours is the use of convolutional neural networks rather than a Transformer model. Despite this difference, their deep learning model is comparable to the one presented in our work. In their test set, they achieved an *R-squared* of 0.44, albeit they opted to use only one substrate for each reaction instead of a reaction fingerprint. Additionally, they randomly selected entries for the test set from the entire dataset, considering the possibility of duplicate structures between the training and test sets. As mentioned in our methodological pipeline and the work of Kroll and Lercher (2023), this approach has the potential to improve models’ performance.

In another study conducted by Kroll et al. (2021), a model was developed for the Michaelis–Menten constant (*K*_m_) using a methodology similar to ours. Initially, when the model solely relied on molecular fingerprints, the coefficient of determination was recorded at 0.40. However, upon incorporating enzyme representations—utilizing the model by Alley et al. (2019) for enzyme representations—the performance improved, achieving an *R-squared* of 0.45 on unseen data points. Furthermore, when only known structures were included in the test set, the *R-squared* achieved was 0.53.

In conclusion, there are no existing works in literature specifically focused on directly calculating the Michaelis–Menten *V*_max_. However, by leveraging studies that indirectly estimate *V*_max_ and employing similar methodologies, the current results are comparable, establishing a reliability framework for the developed models. Importantly, it is worth noting that no other studies were identified in the literature that utilized validation sets in addition to training and test sets. Indeed, while this approach is expected to enhance model performance during training, it does come at the expense of reducing the number of data entries available in the training set. The Keras-based architecture offers model developers the flexibility to seamlessly integrate validation sets into their training procedures.

### 3.9 Study limitations

Enzyme kinetics prediction holds immense promise across numerous domains, including drug discovery, biotechnology, systems biology, and computational New Approach Methodologies (NAMs). In recent years, artificial intelligence techniques have emerged as powerful tools for predicting enzyme kinetics, offering high accuracy, and promising outcomes. In this study, a deep learning methodology capable of estimating *V*_max_ using information derived exclusively from SABIO RK is introduced. Our approach leveraged wild-enzyme representations, reaction fingerprints, and amino acid proportions of the enzyme structure as predictors. Mutated enzymes were not considered in none of the datasets employed. While the developing models yielded auspicious results, it is essential to acknowledge and address certain limitations identified during their development. Addressing these weaknesses has the potential to yield more promising outcomes and enhance the robustness of the models.

Firstly, it is important to note that *V*_max_ does not exclusively rely on the enzyme structure but also on the bioavailable enzyme concentration within a biological system. During the training process concentration data was not considered. This intentional action was taken due to the potential implications of developing models in initializing enzymatic concentrations within a biological system, which could yield valuable insights for computational New Approach Methodologies (NAMs). If concentrations are utilized as predictors, the developing models would become ineffective.

To comprehensively integrate all available biological insights (i.e., structure and bioavailable concentration), one potential approach is to incorporate concentration-response data into the neural network. However, to our knowledge, such data is not extensively cataloged compared to the wealth of information available in databases such as SABIO RK or BRENDA. Despite this concentration gap, by leveraging existing models in the literature and recognizing the proportional relationship between *V*_max_, turnover number, and Michaelis–Menten constant (*K*_m_), the presented models demonstrated consistent performance, affirming the reliability of our approach and methodologies.

It is important to acknowledge the limitations imposed by the small datasets used in our study. The Michaelis–Menten *V*_max_ is a quantity that can be indirectly approximated for an in vitro system, rendering it the least commonly chosen option for direct determination. However, it can be estimated using a *k*_cat_ deep learning model from literature along with enzyme concentration. Due to this fact, the final dataset consisted of less than 1000 unique entries, which is relatively small compared to datasets for turnover numbers or Michaelis–Menten *K*_m_. For instance, datasets exclusively sourced from SABIO RK can exceed 2000 entries. Given that, the models developed experienced lower exposure to new structures which could potentially improve the performance of the developing models. Another significant factor that contributed to the reduction of rows was the absence of .mol files for all compounds in the dataset. These files were utilized for generating molecular fingerprints. If it was unfeasible to manually retrieve a .mol file from KEGG or ChEBI, the corresponding entry was removed. Last but not least, it should also be considered that the selection of specific units (mol/(s*g)) for the datasets resulted in the further reduction of available information.

## 4 Conclusions

Artificial intelligence applications have begun to be applied in all scientific fields with very significant achievements (Akinosho et al., 2020; Hou et al., 2021). In the field of systems biology, cell physiology, and pharmacometrics, biokinetics, toxicokinetics, systems pharmacology and toxicology as well as in the field of life sciences a plethora of innovative approaches have been published so far (Chaturvedula et al., 2019; Cheng et al., 2022; Goßen et al., 2023). These innovative approaches have addressed numerous challenges and gaps offering reliable and robust solutions while guiding research towards targeted endpoints. Importantly, AI has played a crucial role in reducing the need for animal experimentation and conserving resources (Hartung, 2023). One of these fields concerns the determination of kinetic parameters of enzymes such as turnover numbers, Michaelis–Menten constant (*K*_m_) or maximal velocity.

In present work, a custom algorithm that organizes data sourced from SABIO-RK was developed. As a result, three randomly selected datasets were introduced: one for training the model, comprising 70 % of the total dataset entries; another for validation during the training process, consisting of 10 % of the total dataset entries; and one for testing the developed model, which includes 20 % of the total dataset. We also carefully took into consideration the principle introduced by Kroll and Lercher (2023) regarding the fundamental uniqueness of each dataset. Consequently, all subsets contained unique amino acid structures and thus unique entries. To further testing the developing models, a fourth testing dataset considered which included duplicated structures from the training set with different reaction fingerprints. The resulting datasets include numeric enzyme representations, PROTR amino acid proportions and four estimated reaction fingerprints including RCDK standard fingerprints (1024 bits), MACCS keys (166 bits), PubChem fingerprints (881 bits), and E-States fingerprints (79 bits) while the developing models trained to predict *V*_max_. All datasets are available on the GitHub page.

Multiple fingerprints were utilized to find out if the number of bits per molecular structure and therefore per chemical reaction is significant and should be taken into consideration. From the model outputs, it seems that the molecular fingerprint with 1024 bits had a slightly better performance statistically compared to the rest, but the observed differences cannot be considered as significant. As a result of that further testing is needed to ensure that the number of bits does not affect the performance of the model by including more information in the artificial neural network and testing more fingerprints with a higher number of bits.

The protein structure was integrated into the neural network using natural language processing models, specifically by adopting the model introduced by the Facebook Research Team (Rives et al., 2021). In this approach, the amino acid composition was treated as word, resulting in meaningful representations that provided valuable information about the enzyme structure within the deep learning model. This methodology has demonstrated its ability to offer fundamental insights regarding the structural information of proteins (Brandes et al., 2022; Elnaggar et al., 2021; Goldman et al., 2022; Kroll et al., 2022b; Rives et al., 2021). Additionally, an investigation was also conducted to determine the extent to which the ratios of amino acids within the enzymes could serve as reliable predictors for predicting Michaelis–Menten maximal velocity, ultimately yielding a negative conclusion.

The deep learning models were constructed with the utilization of the sequential API of Keras, a top-tier artificial intelligence framework built on TensorFlow. However, to ensure the introduction of reliable models, an in-house code specifically designed to optimize each model was developed. This methodological pipeline leveraged the functionalities provided by the Keras Tuner library (Pon and Krishna Prakash, 2021), ultimately resulting in the development of robust models wherever feasible. It is worth noting that during the optimization process, a trend was observed: as the number of neurons decreased towards the final layer, the performance of the model improved, resulting in more reliable predictions.

The results of the trainings conducted yielded two models with highly valuable metrics compared to those available in the literature. Specifically, the model trained exclusively on enzyme representations achieved an *R-squared* of 0.45 on the unknown structures and 0.70 on the similar ones. The higher performance recorded on known structures confirms the close relationship that *V*_max_ has with the enzyme structure. Subsequently, when reaction fingerprints were included in the neural network, the model's performance did not improve significantly, recording an *R-squared* of 0.46 on the unknown structures and 0.62 on the known ones. The slight decrease in performance on known structures may be attributed to the fact that while the structures are known to the model, the reactions are not. Additionally, the additional complexity to the model led to a decrease in the relative *R-squared*, which was relatively lower compared to the model trained exclusively on enzyme representations.

At this point, it is worth mentioning that the choice of models in the present work is clearly related to the task at hand. Specifically, if the reversibility of reactions and similar enzyme structures catalysing different reactions are not important for the user of the model, the model trained exclusively on ESM-1b representations can be utilized. However, in systems where enzyme structures catalyse more than one biochemical reaction, which may be reversible, the use case of the model incorporating reaction fingerprints is proposed. For this reason, both models are provided on the GitHub page.

Additionally, it is important to emphasize, as noted by Kroll and Lercher (2023), that the development of such models should be based on unique protein structures. This is because identical structures tend to catalyse reactions at comparable rates close to the geometric mean of a reaction's *V*_max_. This pattern was also observed in the present study, where models trained on known structures exhibited significantly better performance compared to those applied to unknown structures. However, the model's performance on unknown structures highlights its high generalizability in estimating Michaelis–Menten *V*_max_ for identical structures catalysing different reactions, as well as for entirely novel structures. As previously mentioned, in both cases, the model demonstrated highly reliable performance.

The development of computational tools, such as the one presented in this work, ensures the estimation of the *V*_max_ value in metabolic conversions without the need for experiments. Additionally, by leveraging computational tools such as the one presented here, along with others available in the literature (such as AI models for approximating turnover numbers), it becomes feasible to determine the concentration of an enzyme in a system using an in-silico approach. This is particularly significant in the development of mechanistic models that quantitatively link genomic information with metabolic information through the inclusion of enzyme concentration in the Michaelis–Menten equation. It is understood that *V*_max_ is not solely dependent on enzyme structure but also on its bioavailable concentration. However, the performance of the models presented is directly comparable to others in the literature, establishing the reliability of the methods used. Furthermore, the utilization of advanced and reliable IVIVE (In Vivo In Vitro Extrapolation) methods can ensure the applicability of the present model to in vivo models as well as humans.

In conclusion, it is worth noting that if enzyme concentration-*V*_max_ response data could be collected on a large scale at some point, it would be worthwhile to finely tune the model to incorporate all the important biological information into the neural network. However, it is important to clarify that the result of developing such a model would not be a tool for initializing the concentration of an enzyme in a system but exclusively predict the Michaelis–Menten *V*_max_.

Indeed, the field of artificial intelligence is experiencing significant growth, with its applications expanding at a rapid pace. Advancements in AI technology have resulted in the development of more reliable and robust models, employing numerous architectures and methodologies to address diverse problems. One of the remarkable aspects of AI is its ability to generate new data that can be used directly or to target specific experiments. Furthermore, data generation capabilities of AI are not limited to merely replicating existing data. It can explore and extrapolate from the provided datasets, creating novel data points that can aid in discovering new patterns, relationships, and potential avenues for further research. This process is particularly valuable when exploring complex and high-dimensional spaces where traditional data collection methods may be limited. Because of these advancements, there is a global trend towards reducing reliance on certain types of experiments, notably those involving laboratory animals or targeted drug testing. Ethical concerns surrounding animal testing have prompted the scientific community to explore alternative approaches that minimize animal involvement. AI-powered simulations and virtual experiments offer a viable and increasingly sophisticated alternative, reducing the need for physical animal experiments while providing valuable insights into various scientific domains. Moreover, the development and investment in more efficient AI algorithms have accelerated the adoption of AI across industries and scientific disciplines. A well-established paradigm regards applications of AI in the Industry 4.0 (Ahmad et al., 2022; Javaid et al., 2022) or the data-driven development of Safe and Sustainable by Design (SSbD) chemicals and materials (Caldeira et al., 2022). The ability of AI to process and analyse vast amounts of data at incredible speeds has transformed the way researchers approach complex problems, leading to faster discoveries and breakthroughs.

## Funding

We acknowledge funding for OBERON (https://oberon-4eu.com/) from the European Union's Horizon 2020 research and innovation program under grant agreement #825712. This publication reflects only the authors’ views, and the European Commission is not responsible for any use that may be made of the information it contains.

## Data availability

The models trained on ESM-1b representations, as well as the one trained on enzyme representations and RCDK fingerprints, can be retrieved from the GitHub page (https://github.com/auth-envelab/VmaxEstimation). Additionally, manually collected datasets from SABIO RK, the processed dataset before splitting, and training, validation, and test indices are also provided.

## CRediT authorship contribution statement

**Achilleas Karakoltzidis:** Writing – review & editing, Writing – original draft, Visualization, Validation, Methodology, Investigation, Formal analysis, Data curation, Conceptualization. **Spyros P. Karakitsios:** Writing – review & editing, Writing – original draft, Supervision, Project administration, Methodology, Investigation, Conceptualization. **Dimosthenis Α. Sarigiannis:** Writing – review & editing, Writing – original draft, Supervision, Resources, Project administration, Methodology, Funding acquisition, Conceptualization.

## Declaration of competing interest

The authors declare that they have no known competing financial interests or personal relationships that could have appeared to influence the work reported in this paper.

## Footnotes

1 https://eagerai.github.io/kerastuneR/
